# Methodological Challenges and Opportunities in Population Aging Research

**DOI:** 10.1093/geroni/igaf122.1854

**Published:** 2025-12-31

**Authors:** Eleanor Hayes-Larson, Ruijia Chen, Delany Salazar

## Abstract

Population health research on aging is complicated by methodological issues such as measurement challenges and error, competing risks, and selective attrition, each of which may manifest in multiple ways as sources of bias and affect reproducibility of results. Novel methods have been developed to address these issues. The session features cutting-edge research that incorporates novel epidemiological methods to address key methodological challenges in population aging research. Speakers will address (1) measures to crosswalk between cognitive instruments to better compare results across studies, (2) use of simulation to understand sources of bias such as measurement error in biomarker analyses, (3) the impact of mortality as a competing risk in aging-related causal questions, (4) approaches to account for inter-wave mortality in cohort studies with high mortality, and (5) use of multiverse analysis to understand importance of operational definition of constructs like social isolation for replication of study results.

